# Kinome expression profiling and prognosis of basal breast cancers

**DOI:** 10.1186/1476-4598-10-86

**Published:** 2011-07-21

**Authors:** Renaud Sabatier, Pascal Finetti, Emilie Mamessier, Stéphane Raynaud, Nathalie Cervera, Eric Lambaudie, Jocelyne Jacquemier, Patrice Viens, Daniel Birnbaum, François Bertucci

**Affiliations:** 1 Department of Molecular Oncology, Centre de Recherche en Cancérologie de Marseille, UMR891 Inserm, Institut Paoli-Calmettes, 27 bd Leï Roure, 13009 Marseille, France; 2 Department of Medical Oncology, Institut Paoli-Calmettes, 232 Bd. Ste-Marguerite, 13273 Marseille Cedex 09, France; 3 Centre d'Immunologie Marseille-Luminy, Parc Scientifique & Technologique de Luminy - Case 906 - F13288 Marseille cedex 09, France; 4 Department of Surgery, Institut Paoli-Calmettes, 232 Bd. Ste-Marguerite, 13273 Marseille Cedex 09, France; 5 Department of Biopathology, Institut Paoli-Calmettes, 232 Bd. Ste-Marguerite, 13273 Marseille Cedex 09, France; 6 Université de la Méditerranée, 58 Bd Charles Livon, 13284 Marseille Cedex 07, France

**Keywords:** breast cancer, basal-like, gene expression profiling, prognosis, immune response

## Abstract

**Background:**

Basal breast cancers (BCs) represent ~15% of BCs. Although overall poor, prognosis is heterogeneous. Identification of good- *versus *poor-prognosis patients is difficult or impossible using the standard histoclinical features and the recently defined prognostic gene expression signatures (GES). Kinases are often activated or overexpressed in cancers, and constitute targets for successful therapies. We sought to define a prognostic model of basal BCs based on kinome expression profiling.

**Methods:**

DNA microarray-based gene expression and histoclinical data of 2515 early BCs from thirteen datasets were collected. We searched for a kinome-based GES associated with disease-free survival (DFS) in basal BCs of the learning set using a metagene-based approach. The signature was then tested in basal tumors of the independent validation set.

**Results:**

A total of 591 samples were basal. We identified a 28-kinase metagene associated with DFS in the learning set (N = 73). This metagene was associated with immune response and particularly cytotoxic T-cell response. On multivariate analysis, a metagene-based predictor outperformed the classical prognostic factors, both in the learning and the validation (N = 518) sets, independently of the lymphocyte infiltrate. In the validation set, patients whose tumors overexpressed the metagene had a 78% 5-year DFS *versus *54% for other patients (p = 1.62E-4, log-rank test).

**Conclusions:**

Based on kinome expression, we identified a predictor that separated basal BCs into two subgroups of different prognosis. Tumors associated with higher activation of cytotoxic tumor-infiltrative lymphocytes harbored a better prognosis. Such classification should help tailor the treatment and develop new therapies based on immune response manipulation.

## Background

Breast cancer (BC) is heterogeneous. Gene expression profiling has identified molecular subtypes with different biological features and different outcome [1-5], including basal BCs. Basal BCs, which represent ~15-20% of invasive BCs are high-grade tumors, frequently do not express hormone receptors (HR) and ERBB2, and have the worst prognosis overall [6,7]. Yet, basal tumors show prognostic heterogeneity. Both the standard histoclinical features and the recently defined prognostic gene expression signatures (GES) fail to identify patients who will relapse and patients who will not respond to chemotherapy [8]. Defining the molecular bases of this heterogeneity should help better understand these tumors, identify new therapeutic targets and more reliable predictors of survival and therapeutic response.

Kinases, which constitute ~1.7% of human genes [9], are activated or overexpressed in cancers [10], and constitute current or future targets for successful therapies [11]. Previously, we identified a 16-kinase GES that improved the prognostic classification of luminal BCs [12]. A similar approach was successfully applied to 44 estrogen receptor (ER)-negative BCs, including ERBB2-positive tumors and less than 50% of basal tumors [13]. To our knowledge, the "kinome approach" has never been applied to basal BCs only.

We tested the hypothesis that the expression of kinase genes may distinguish good- from poor-prognosis basal tumors.

## Methods

### Patients' selection

Institut Paoli-Calmettes (IPC) and public retrospective data sets from BC samples profiled using oligonucleotide microarrays were collected (Additional file 1, Table S1). All were pre-treatment samples of invasive non-inflammatory and non-metastatic adenocarcinomas. Microarray data from our set are available through Gene Expression Omnibus (series entry GSE21653).

The "IPC" training set included 261 patients who underwent initial surgery in our institution between 1992 and 2007. Each patient gave written informed consent and this study has been approved by our institutional ethics committee. All samples were histologically reviewed in a standardized fashion by a pathologist (JJ) to asses the ER, progesterone receptor (PR) and ERBB2 status by immunohistochemistry (IHC), and the percent of cancer cells (superior to 60%). Antibodies used (Dak^®^) were SP1 clone for ER, PgR636 clone for PR and Herceptest™ for ERBB2. The cut-off for positivity was 1% of stained tumor cells for HR, and ERBB2 status (0-3+ score, DAKO HercepTest kit scoring guidelines) was defined as positive if 3+ or 2+ with amplification confirmed by in situ hybridization. Vascular invasion and lymphocytic infiltration were assessed by Hematoxylin and Eosin Staining (HES).

Twelve pooled public data sets constituted the validation set including a total of 2254 samples [5,7,14-23]. DFS was the best annotated survival information among these sets and was chosen as survival end-point.

### Gene expression data analysis

Details are given in Additional file 2 (Supplementary Material). Data sets were processed as previously described [24]. Briefly, for the Agilent sets, we applied quantile normalization to available processed data. Regarding the Affymetrix sets, we used Robust Multichip Average (RMA) with the non-parametric quantile algorithm as normalization parameter [25]. Quantile normalization or RMA was done in R using Bioconductor and associated packages. The five molecular subtypes were determined using the single sample predictor (SSP) classifier [26].

Other analyses were centered on 771 kinase and kinase-interacting genes, based on an update of the initial kinome description [9,13]. This list was matched with genes available on the Affymetrix U133 Plus 2.0 microarrays used to profile the IPC tumor set, finally retaining 661 genes (Additional file 3, Table S2). Analyses were both unsupervised and supervised. Supervised t-test analysis searched for genes upregulated in basal samples compared to at least one of the four other molecular subtypes, with 5% significance and a false discovery rate (FDR) lower than 5%. To circumvent the problem of dissymmetry of variables with a number of samples inferior to the number of genes being tested [14,27-31], we grouped the resulting genes with correlated and interdependent expression (gene subsets) in a single "metagene". Metagene expression value is the mean of the normalized expression values of all genes in the respective gene subset. Each metagene is treated as if it were a single gene, thereby reducing data dimension. We defined our metagenes by hierarchical clustering using data median-centered on genes, Pearson correlation as similarity metrics and centroid linkage clustering [32]. We identified robust gene clusters (minimal cluster size and minimal Pearson correlation were 15 and 0.6, respectively) using the quality-threshold (QT) clustering method [32]. A metagene was then computed for each selected cluster, and its prognostic incidence (as continuous value) evaluated using a Cox regression univariate analysis. Once a metagene associated with DFS (5% level significance) was defined, its expression value was used to divide the training set into two subgroups then tested for association with DFS. The cut-off was defined as the best threshold dividing the population into two subgroups with the greater DFS difference, "Metagene-Low" (expression value inferior to the threshold) and "Metagene-High" (expression value above) subgroups. This cut-off was applied to basal tumors of each validation series, and the define subgroups were then pooled before prognostic analysis.

We tested the prognostic value of two recently reported classifiers associated with survival in basal BCs: the medullary BC (MBC) classifier [33] and the HER2-derived prognostic predictor (HDPP) [34] associated with survival in both ERBB2+ and basal tumors. We also tested three multigene signatures identified as prognostic in breast cancer, independently of molecular subtypes: the Genomic Grade Index [16], the 76-gene signature [15], and the 70-gene signature [5]. Ontology analysis was done using Ingenuity Pathway Analysis (IPA) software (Redwood City, CA, USA) [35]. We also determined if immune signatures [36] were overrepresented in one prognostic subgroup using the gene set enrichment analysis (GSEA) algorithm and 1000 permutations [37].

### Statistical analysis

Correlations between sample groups and histoclinical factors were calculated with the Fisher's exact test and the t-test when appropriate. DFS was calculated from the date of diagnosis until date of first relapse or death using the Kaplan-Meier method, and follow-up was measured to the date of last news for event-free patients. Survival curves were compared with the log-rank test. Univariate and multivariate prognostic analyses used the Cox regression method. Univariate analyses tested classical histoclinical factors: age (≤50 years *vs*. > 50), pathological tumor size (pT≤20 mm *vs*. > 20), lymph node status (pN positive *vs*. negative), SBR grade (I *vs*. II-III), IHC ER status (negative *vs*. positive), peritumoral vascular invasion (negative *vs*. positive) and lymphocytic infiltrate. Data regarding the delivery of adjuvant chemotherapy and hormone therapy were also analyzed. Analyses included also binary classifications based on the immune metagene, the MBC and HDDP classifiers (good *vs*. poor-prognosis subgroups). Multivariate analyses tested all variables with a p-value inferior to 0.05 in univariate analysis and excluded patients with one or more missing data. All statistical tests were two-sided at the 5% level of significance. Analyses were done using the survival package (version 2.30), in the R software (version 2.9.1). Our analysis adhered to the reporting recommendations for tumor marker prognostic studies (REMARK) [38].

## Results

### Identification of a prognostic kinase expression signature

Five hundreds and ninety-one out of 2515 tumors were basal, including 73/261 in our IPC series and 518/2254 in the public sets (Table 1). These tumors displayed classical histoclinical features of basal BC (Additional file 4, Table S3). Clinical outcome, available for 2109 patients, correlated with subtypes with 5-year DFS of 83% for luminal A, 60% for luminal B, 77% for normal-like, 61% for basal, and 61% for ERBB2-overexpressing.

**Table 1:** Histoclinical features of basal-like tumors (IPC and validation series)

Characteristics (N)	Basal
	N = 591
	N (% of evaluated cases)
Age (445)	
≤ 50 years	215 (57%)
> 50 years	162 (43%)
Histological type (256)	
ductal	234 (91%)
lobular	7 (3%)
other*	15 (6%)
Pathological tumor size, pT (466)	
pT1	115 (25%)
pT2-4	351 (75%)
Pathological lymph node status, pN (493)	
negative	314 (64%)
positive	179 (36%)
Tumor grade (493)	
SBR 1	14 (3%)
SBR 2-3	479 (97%)
IHC ER status (507)	
negative	411 (81%)
positive	96 (19%)
IHC PR status (223)	
negative	199 (89%)
positive	24 (11%)
IHC ERBB2 status (105)	
negative	86 (84%)
positive	19 (16%)
Adjuvant chemotherapy (309)	
no	203 (66%)
yes	106 (34%)
Adjuvant hormone therapy (322)	
no	237 (95%)
yes	13 (5%)
Events (453)**	183 (40%)
5-year DFS (453)**	61%

*4 metaplastic carcinomas, 4 mixed adenocarcnomas, 1 mucinous carcinoma, and 5 adenocarcinomas non otherwise specified. **out of these 453 patients with available follow-up, 193 did not received any systemic adjuvant treatment, 115 received adjuvant systemic therapy, no patient received adjuvant Trastuzumab, and data were unavailable for 145 patients.

**Figure 1:**
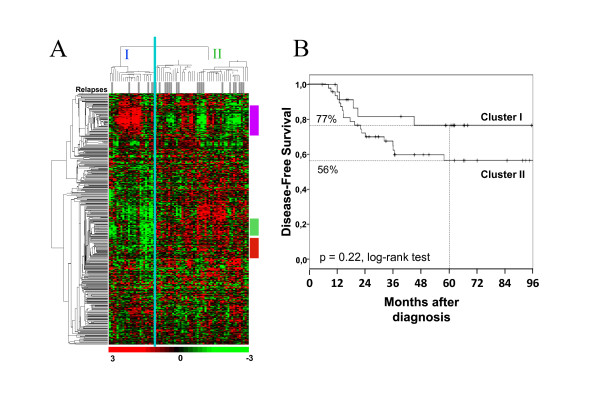
**Hierarchical clustering of basal breast cancer**. (A) Unsupervised hierarchical clustering of 73 non-metastatic non-inflammatory basal BCs from IPC with 360 genes coding for kinase or kinase-interacting proteins overexpressed in basal tumors. Each row represents a gene and each column a sample. The expression level of each gene in each sample is relative to its median abundance across the samples and is depicted according to the color scale shown under the matrix. Red and green indicate expression levels respectively above and below the median. Relapses are indicated in the stripe under the dendrogram: white for no relapse during follow-up, and grey for relapse. Two tumor clusters (I and II) are delineated by the vertical green line. To the right, vertical colored bars indicate the three clusters identified by the QT clustering method: purple, immune-related cluster; green, biologically unspecific cluster; red, proliferation-related cluster. (B) Kaplan-Meier disease-free survival curves for cluster I patients (n = 24), and cluster II patients (n = 49).

The 73 IPC basal tumors were used as training set for identifying a prognostic kinase GES from the 661-gene list. Supervised analysis identified 581 genes differentially expressed in basal *versus *at least one other subtype, including 360 genes overexpressed in basal tumors (Additional file 3, Table S2). Within this series most of the patients (90%) received adjuvant chemotherapy. Twenty-five patients developed relapse or death with a median time-to-relapse of 19 months, and forty-eight patients remained disease-free with a median follow-up of 64 months. The 5-year DFS was 63%. Hierarchical clustering of these tumors with the 360-gene set (Figure 1A) revealed two main clusters, I (n = 24) and II (n = 49), with 5-year DFS superior in cluster I (77% *versus *56%; p = 0.22, log-rank test; Figure 1B). QT clustering identified three gene clusters with a major role in this discrimination (Figure 1A, and Additional file 5, Table S4). One included 21 genes not related to any specific biologic function. A second cluster was associated with the cell cycle. The third cluster (thereafter designed immune cluster) contained 28 genes, which for many were involved in immune signaling (e.g. *BLK, BTK, FYN, SYK, ITK, JAK3, LCK, LCP2, PRKCB, and ZAP70*). Visually, lower expression of this cluster was associated with more relapses (Figure 1A). We built a metagene for each gene cluster, and analyzed their correlation with DFS using univariate Cox regression analysis. Only the immune metagene correlated with DFS (HR = 0.32, 95%CI [0.17-0.68], p = 2.4E-3, Wald test; Additional file 6, Table S5). Resampling with 100,000 iterations showed only a 0.8% probability to find a metagene built from 28 random genes with similar or better prognostic correlation than the immune metagene.

**Figure 2:**
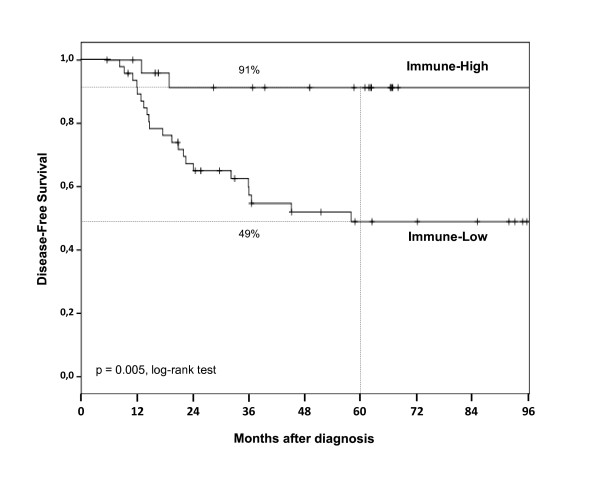
**Disease-free survival and basal subgroups in the learning set**. Kaplan-Meier disease-free survival curves of basal BC patients in the IPC series according to the subgroups "Immune-High" (n = 25) and "Immune-Low" (n = 48).

We defined two subgroups of basal tumors according to the immune metagene expression value: "Immune-High" if above the value threshold (n = 25) and "Immune-Low" if under (n = 48). No histoclinical factor including the lymphocyte infiltrate was different between the two subgroups (Additional file 7, Table S6). Survival was different, with 91% 5-year DFS in "Immune-High" subgroup *versus *49% in "Immune-Low" (p = 0.005, log-rank test, Figure 2). On univariate analysis (Table 2), two factors were associated with DFS: vascular invasion (HR = 2.32, 95%CI [1.04-5.18], p = 0.04, Wald test) and immune metagene expression (HR = 0.21, 95%CI [0.06-0.70], p = 0.01, Wald test). They remained significant on multivariate analysis (Table 2).

**Table 2:** Univariate and multivariate analyses by Cox regression of basal tumors, IPC series

		Univariate Analysis			Multivariate Analysis	
	
	N	HR [95% CI]	*p*-value	N	HR [95% CI]	*p*-value
**Age ≤50 (vs > 50 y)**	73	1.83 [0.79-4.26]	0.16			
**pT > 20 mm (vs ≤ 20 mm)**	73	1 [0.96-1.05]	0.95			
**pN pos (vs neg)**	73	1.93 [0.88-4.24]	0.1			
Grd 2-3 (vs 1)*	73	0.15 [0.02-1.18]	0.07			
**ER pos (vs neg)**	73	1.08 [0.25-4.68]	0.91			
**Vascular invasion **	72	2.32 [1.04-5.18]	**0.04**	72	2.30 [1.03-5.14]	**0.04**
Lymphocyte infiltrate **	71	0.38 [0.11-1.28]	0.12			
**Chemotherapy**	73	0.62 [0.18-2.12]	0.62			
**Hormone therapy**	72	1.76 [0.64-4.81]	0.27			
**Immune metagene High (vs Low)**	73	0.21 [0.06-0.70]	**0.01**	72	0.22 [0.07-0.73]	**0.01**

* Only 1 tumor was grade 1

**absent to low vs moderate to high.

We also performed a similar analysis on genes underexpressed in basal tumors, but it did not allow the identification of any robust gene clusters.

### Validation of the prognostic signature

**Figure 3:**
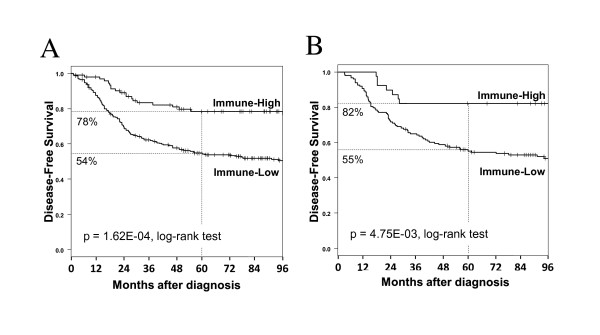
**Disease-free survival and basal subgroups in the validation set**. Kaplan-Meier disease-free survival curves of basal BC patients in the independent validation series according to the subgroups "Immune-High" and "Immune-Low". (A) in all patients (95 *versus *285 patients respectively), and (B) in patients having received no systemic adjuvant therapy (39 *versus *148 patients respectively).

The expression of the immune metagene was studied in the independent panel of 518 basal tumors not used to define the predictor. Follow-up for DFS was annotated for 380 patients: 158 developed relapse or death with a median time-to-relapse of 30 months, and 222 remained disease-free with a median follow-up of 93 months. The 5-year DFS was 60%. At least 25 out of 28 (mean = 27) genes included in the immune metagene were common to each separate set (Additional file 1, Table S1). A total of 122 patients were defined as "Immune-High" and 396 as "Immune-Low". Their histoclinical features (including the lymphocyte infiltrate available in 56 out of 518 tumors) were well balanced, except SBR grading, more frequently II-III in the "Immune-High" subgroup (Table 3). The 5-year DFS was 78% in the "Immune-High" subgroup and 54% in the "Immune-Low" one (p = 1.62E-04, log-rank test, Figure 3A), confirming the prognostic value of the immune metagene. Analysis by data set showed that the mean difference of 5-year DFS between "Immune-high" and "Immune-low" cases was 25% (95%CI, [13 - 37], p = 0.0038, T-test).

**Table 3:** Histoclinical comparison of the two basal subgroups defined with the immune metagene in the independent validation series

Characteristics (N)	**Immune-High **n = 122	**Immune-Low **n = 396	*p*-value	OR (95%CI)
	
	N (% of evaluated cases)			
Age (372)			0.71*	
≤ 50 years	53 (62%)	169 (59%)		1
> 50 years	33 (38%)	117 (41%)		1.11 (0.66-1.89)

Pathological tumor size, pT (394)			0.11*	
pT1	32 (33%)	73 (24%)		1
pT2-4	64 (67%)	225 (76%)		1.54 (0.9-2.6)

Pathological lymph node status, pN (420)			0.90*	
negative	62 (65%)	208 (64%)		1
positive	33 (35%)	117 (36%)		1.06 (0.64-1.77)

Tumor grade (420)			ND	
SBR 1	0 (0%)	13 (4%)		
SBR 2-3	103 (100%)	304 (96%)		

IHC ER status (434)			0.57*	
negative	73 (77%)	269 (79%)		1
positive	22 (23%)	70 (21%)		0.86 (0.49-1.57)

Lymphocyte infiltrate (56)			0.51*	
absent	6 (46%)	14 (33%)		1
present	7 (54%)	29 (67%)		1.76 (0.41-7.48)

Adjuvant chemotherapy (354)			0.43*	
no	49 (64%)	162 (58%)		1
yes	28 (36%)	115 (42%)		1.24 (0.72-2.18)

Adjuvant hormone therapy (269)			0.11*	
no	59 (91%)	197 (97%)		1
yes	6 (9%)	7 (3%)		0.40 (0.12-1.46)

Follow-up (months, median) (380)	95	89	0.44**	
Relapses (380)	25 (26.3%)	133 (46.7%)	4.77 E-04*	0.41 (0.23-0.70)
5-year DFS (380)	78%	54%	1.6 E-04***	

N, number of tumor samples - out of the 2515 samples - with available information for the corresponding characteristic, *, Fisher's exact test; **, Mann-Whitney test; ***, log-rank test; ND, not done.

On univariate analysis (Table 4), two factors correlated with DFS: lymph node involvement (HR = 1.53, 95%CI [1.04-2.25]; p = 0.03, Wald test) and immune metagene expression (HR = 0.45, 95%CI [0.29-0.69]; p = 2.9E-04, Wald test). On multivariate analysis, both remained significant.

**Table 4:** Univariate and multivariate (with and without MBC-based classifier) DFS analyses by Cox regression of basal tumors: public series

	Univariate Analysis			Multivariate Analysis*				
	
	N	HR [95%CI]	*p*-value	N	HR [95%CI]	*p*-value	HR [95%CI]	*p*-value
**Age ≤ 50 (*vs *> 50y)**	253	0.96 [0.65-1.41]	0.84					
**pT > 20 mm (*vs *≤ 20 mm)**	275	1.40 [0.93-2.11]	0.11					
**pN pos (*vs *neg)**	301	1.53 [1.04-2.25]	0.032	301	1.58 [1.07-2.33]	0.021	1.46 [0.99-2.16]	0.06
**Grd 2-3 (*vs *1)**	302	3.00 [0.74-12.1]	0.12					
**ER pos (*vs *neg)**	315	0.68 [0.45-1.03]	0.07					
**Chemotherapy**	236	1.28 [0.77-2.14]	0.34					
**Hormone therapy**	250	1.01 [0.41-2.48]	0.98					
**MBC-based classifier**	380	0.59 [0.43-0.82]	1.72 E-04	301			0.59 [0.40-0.87]	7.5 E-03
**Immune metagene High (*vs *Low)**	380	0.45 [0.29-0.69]	2.4 E04	301	2.15 [1.32-3.50]	0.0022	0.54 [0.33-0.89]	0.015

* multavariate analyses were performed without (left) and with (right) the medullary-based classifier.

### Comparison with existing classifiers

Two prognostic multigenic models have been reported in basal BC: the MBC and HDPP classifiers [33,34]. We assessed their prognostic value in the present 518 basal tumors. On univariate analysis, the MBC classifier correlated with DFS, with a HR for relapse of 0.59 (95% CI [0.43-0.82], p = 0.0017) for good-prognosis patients as compared with poor-prognosis patients. In multivariate analysis including this classifier, our immune metagene classifier and lymph node status showed that both genomic classifiers were significant, whereas node involvement was not (Table 4), suggesting that the multigenic models have independent prognostic value. The HDPP classifier confirmed its prognostic value for ERBB2-overexpressing tumors in our series (n = 214), but not in the 518 basal samples: 5-year DFS was 63% for the good-prognosis patients *versus *61% for the poor-prognosis patients (p = 0.62, log-rank test).

We also assessed the prognostic impact of three published major prognostic expression signatures recently reported in early breast cancer. In each data set, each sample was assigned a good or a poor prognosis based on each signature. Data sets were then pooled, and survival was compared between the predicted good-prognosis and poor-prognosis subgroups. Univariate DFS analysis performed in the basal subtype showed that none of these classifiers was associated with survival (Table 5). These results show the absence of informative value of these signatures in the basal subtype, by contrast with our classifier.

**Table 5:** Comparison of the prognostic value of the immune-metagene classifier with three available signatures, Disease-free survival, Cox univariate analysis

		N	HR [IC95]	*p*-value
**Immune-metagene**	High vs Low	380	2.23 [1.45-3.42]	2.4 E-04
**70-gene signature**	Poor vs Good	380	1 [1]	NaN*
**Genomic grade index**	High vs Low	380	1.30 [0.53-3.18]	0.56
**76-gene signature**	Poor vs Good	317	1.40 [0.96-2.03]	0.08

*all basal tumors were classifeid as "poor prognosis" by this classifier.

### Prognostic and/or predictive value of the immune classifier?

To determine the link of the immune metagene with metastatic risk and/or with response to chemotherapy, we analyzed - within the series of 518 basal BCs - the 187 cases with available follow-up who had not received any adjuvant systemic therapy. In this set, "Immune-High" patients had a longer DFS than "Immune-Low" patients with 5-year DFS of 82 *vs*. 55% respectively (p = 4.75E-03, log-rank test; Figure 3B).

Next, we studied the capacity of our model to predict pathological complete response (pCR) after anthracycline-based neoadjuvant chemotherapy. Information was available for two data sets with the following regimens: weekly paclitaxel and fluorouracil-doxorubicin-cyclophosphamide (55 patients with pCR and 70 without) [22], and fluorouracil-epirubicin-cyclophosphamide or docetaxel followed with docetaxel-epirubicin (34 patients with pCR and 99 without) [23]. We identified 98 basal cases out of the 258 included samples. "Immune-High" patients experienced more pCR (59%) than "Immune-Low" patients (43%), but the difference was not significant (Odds ratio = 1.87, 95%CI [0.57-6.40], p = 0.29, Fisher's exact test).

Altogether, these observations suggested that the immune metagene is associated with relapse risk, whereas its association with response to chemotherapy deserves to be tested in larger series.

### The immune kinase metagene correlates with cytotoxic T-cell response

We next sought to elucidate the type of immune response associated with our metagene. Ontology analysis of the 28 genes using IPA software confirmed association with many pathways involved in immune response [35], particularly in lymphocyte activation processes, such as "T-cell receptor signaling", "CD28 signaling in T helper cells", "NK cell signaling", "PLC signaling", "Role of NFAT in regulation of the immune response", "NF-kB signaling", or "IL2 signaling" (Additional file 8 - Table S7, and Additional file 9 - Figure S1). The upregulation of *BTK, CD3E, FYN, ITK, LCK, LCP2, PRKCs, SYK, ZAP70 *and *JAK3 *clearly identified an activated profile of the lymphocytic lineage.

To better explore the molecular differences between "Immune-High" and "Immune-Low" basal BCs, we searched for the genes differentially expressed between the two subgroups in the IPC series using the whole genome and not only the kinome. Supervised analysis (0.1% FDR) identified 532 differential genes. Most of them (n = 506) were overexpressed in "Immune-High" samples (Additional file 10, Table S8A). Ontology analysis showed that these genes were particularly involved in immune response, and more specifically in adaptive immunity (Additional file 10, Table S8B). To confirm this observation, we applied GSEA using reported T-cell, CD8+ T-cell and B-cell expression signatures [36]. As shown in Additional file 11 (Figure S2), an enrichment of genes involved in T-cell, CD8+ T-cell and B-cell signatures was found in "Immune-High" cases.

## Discussion

Basal BCs are poor-prognosis tumors, which require both improvement of our ability to predict the clinical outcome for better tailoring treatment and identification of new therapeutic targets. Their prognosis is heterogeneous, and it is currently impossible to predict which patients will or will not relapse using classical histoclinical factors or the recently reported prognostic GES, notably those currently tested in clinical trials [39]. In the same way, the HDDP classifier [34] identified using ERBB2+ tumors, failed to classify basal samples. Prognostic analyses should be done per subtype [40].

Analysis of kinase and kinase-related genes might help develop new targeted therapies. We report a kinase-based model that divides basal BCs into two subgroups with balanced histoclinical factors but different survival (25% difference for 5-year DFS). This model is based on the expression of an immune 28-gene metagene. Identified in a learning set, its prognostic value was confirmed in an independent data set of 518 cases. The model outperformed the individual current prognostic factors on multivariate analysis, both in the learning and validation sets. Patients with high expression of the immune metagene had a better DFS than other patients. This prognostic value remained when applied to patients treated without any adjuvant chemotherapy, suggesting a link with the metastatic potential. An additional link with chemosensitivity cannot be excluded as "Immune-High" patients experienced a higher, but non significant, pCR rate than "Immune-Low" patients.

The favorable prognostic impact of the immune response, particularly the T-cell response, has been reported in ER-negative [8,13,14,26,41-43] or ERBB2+ BCs [8,28,31,44]. Similar finding was reported in 97 triple-negative BCs [45] in which increased expression of interferon-related genes tended to confer better prognosis. In our previous study [33] and the present one, we focused on basal BC only, since this subtype is even more homogeneous than the triple-negative group [46]. In our previous study, we defined a 368-gene prognosticator, which confirmed the positive influence of T_H_1 cells and high cytotoxic activity. This model outperformed two immune signatures in multivariate analysis of DFS [28,42]. We showed here that both the immune kinase model and this previous model maintain their prognostic value in multivariate analysis, suggesting their independence. It is of note that our "immune-metagene" model presented a prognostic value in luminal B (p = 0.03, Wald test) and ERBB2-overexpressing cases (p = 0.02, Wald test), but not in luminal A and normal-like samples (p = 0.58 and 0.98, respectively, Wald test). Moreover, it is worth noting that previously published signatures (Genomic grade index, 70-gene signature, and 76-gene signature), mainly based on proliferation, failed to separate good from poor prognosis basal breast cancers.

Ingenuity analysis of both the 28 genes and the genes differentially expressed between the two subgroups defined by our kinase immune metagene confirmed that the differences between these histoclinically similar subgroups are in immune genes. Upregulated kinome-genes suggest the presence of an activated lymphocyte infiltrate in "Immune-High" patients. This lymphocyte-activated status is due to stimulations by cytokines (JAK3, STAT1, STAT4, TBX21 and T_H_1 cytokine receptors), by T-cell receptor (T-cell receptor chains [alpha, beta and gamma], CD3E, CD3D, CD247/CD3Z, CD28, CD27, CD2, CD8A, CD4, LAG3, MAL, LAT2, PIM2), by B-cell receptor (CD19, CD79b, CD27, CD40, IGJ, IGK@, IGH@, BTK, BLNK, BANK1), and by anti-tumor receptors (KLRK1, KLRB1, GAB3, SLAMF1, SLAMF6-8). The lymphocyte infiltrate is strictly T_H_1-biased with the overexpression of *IL2RG, IL23RB *and *IL7R *involved in lymphocyte survival, of *IL12RB1, IL15RA, IL18BP*, and *IL21R *T_H_1-biased receptors, of *STAT1, STAT4*, and *TBX21 *T_H_1 transcription factors, and of several interferon-inducible molecules (*GVIN1, ISG20, GBP2, IRF1, IRF4, IRF7, and IRF8*). This agrees with increased levels of cytotoxic granules and pore-forming molecules (VAMP1, GZMA, GZMB, GZMH, GZMK, GNL, PRF1, CFLAR, CASP1, and CASP10). Interestingly, there are also several genes encoding activated memory lymphocyte recruitment such as IL16, XCL1, CCL5, CCR5, CXCL9, CXCR3, CCL19, CCR7, and CXCR6 (mostly helper and cytotoxic T-cells), and CXCL13, CXCR5 (activated B-cells), among which some are strictly produced by activated T-cells, such as CCL4 and CCL5. Finally, we also found transcripts involved in lymphocyte migration and/or activation (ITGAL and ITGB2 heterodimers, ITGA4, ITGAX, ITGB7, SELL, SELP, SELPL, and CD69).

Thus, we show that the immune response, and notably the adaptive cytotoxic T_H_1-cell response [47], influence survival of basal BC patients. Despite the small size of the independent population with lymphocyte infiltrate data available, which does not allow to really conclude about the impact of the quantity of lymphocyte infiltrate on the expression of immune response-related genes, the absence of correlation between the immune metagene and lymphocyte infiltration in our cohort and in two independent data sets [5,8] as well as the function of genes, suggest that this influence does not depend on the degree of lymphocyte infiltrate, but on the efficiency of its cytotoxic activation status. The differential expression of these "immune genes" is probably also due to a variable expression of epithelial-derived molecules [13,42,48], which activate (in "Immune-High" cases) or repress (in "Immune-Low" cases) the local immune response to the tumor. These hypotheses deserve further investigation to understand the respective role of tumor-infiltrative lymphocytes and cancer cells on cancer history.

## Conclusions

In conclusion, we propose a robust prognostic subdivision of basal BC based on the expression of 28 genes, involved in immune response and notably the cytotoxic T-cell response. Tumors associated with higher activation of cytotoxic tumor-infiltrative lymphocytes have a better prognosis, and are likely to better respond to chemotherapy. Such classification should help tailor treatment. Furthermore, since adaptive immunity seems to play a pivotal role [49] immune response manipulation might be an efficient way of treating or preventing these poor-prognosis tumors [47,50].

## Abbreviations

BC: breast cancer; DFS: disease-free survival; ER: estrogen receptor; FDR: false discovery rate; GES: gene expression signature; GSEA: gene set enrichment analysis; HES: Hematoxylin and Eosin Staining; HDPP: HER2-derived prognostic classifier; HR: hormone receptor; IHC: immunohistochemistry; IPA: Ingenuity Pathway Analysis; IPC: Institut Paoli-Calmettes; MBC: medullary breast cancer; PR: progesterone receptor; QT: quality-threshold; RMA: robust multichip average; SSP: single sample predictor.

## Competing interests

The authors declare that they have no competing interests.

## Authors' contributions

RS and FB designed the concept of the study. FB, EL and PV were responsible for provision of study patients. JJ was responsible for pathologic examination. RS, PF and NC were responsible for samples and data gathering, nucleic acids extraction and microarray experiments. RS, PF and FB were responsible for statistical analysis and interpretation. RS, FB and DB wrote the final manuscript. All authors read and approved the final manuscript.

## Supplementary Material

Additional file 1 **Table S1: Description of the breast cancer data sets**. Click here for file

Additional file 2 **Supplementary materials**. Click here for file

Additional file 3 **Table S2: List of 661 kinase and kinase-related genes analyzed**. Click here for file

Additional file 4 **Table S3: Molecular subtypes and histoclinical features of the pooled data sets**. Click here for file

Additional file 5 **Table S4: Description of the three genes clusters identified with QT clustering**. Click here for file

Additional file 6 **Table S5: Univariate DFS analysis of metagenes by Cox regression of basal tumors: IPC series**. Click here for file

Additional file 7 **Table S6: Histoclinical comparison of the two basal subgroups defined with the immune metagene (IPC series)**. Click here for file

Additional file 8 **Table S7: Ingenuity canonical pathways associated with the immune-related cluster**. Click here for file

Additional file 9 **Figure S1: Biological network of genes included in or associated with our 28-gene model**. A fine-tuning between inhibitor (phosphatases) and activator (kinases) signals regulates lymphocyte anti-tumor immunity. AK and Pyk2 are two of the major kinases that become tyrosine phosphorylated following lymphocyte stimulation. Both are associated to Lck. Lck (lymphocyte specific kinase) and Fyn are cytoplasmic tyrosine kinases of the Src family expressed in T-cells and natural killer (NK) cells, under the T cell receptor (TCR) or Natural cytotoxicity receptor (NCR). Their activity is critical for T and NK cell receptors-mediated signaling, leading to normal T- and NK-cell development and activation. Increased Fyn transcript and protein content in T cells can be observed with high T cell activity. Square 1. LAT is a linker protein essential for activation of T lymphocytes. Its rapid tyrosine-phosphorylation upon TCR stimulation recruits downstream signaling molecules for membrane targeting and activation. LAT is a substrate for Syk/Zap70 kinase and an immediate substrate for both Lck and Syk kinases. Its phosphorylation is an early event leading to T-cell activation. Both Lck and Syk phosphorylate the ITAM-like motifs on LAT, which is essential for induction of the interaction of LAT with downstream signaling molecules such as Grb2, PLC-γ1 and for activation of MAPK-ERK pathways. ZAP70 is thus at the crossroad of several signaling pathways that control lymphocyte development and function and cell survival in response to a wide variety of activator signals coming from the NCR, TCR or other receptor involved in anti-tumor immunity. Square 2. Cytokines receptors express at the membrane also regulate lymphocyte activation through the JAK-STAT signaling pathway. Square 3. In B, T and NK cells, the inhibition of these kinases is mostly mediated by protein tyrosine phosphatases (PTP), regrouping members of the SHP family (SHP-1, SHP-2) or LYP family. These proteins inhibit effector phase functions by dephosphorylating a wide spectrum of phospho-proteins involved in hematopoietic cell signaling. Click here for file

Additional file 10 **Table S8: Genes differentially expressed between the "Immune-High" and "Immune-Low" basal tumor subgroups in the IPC set**. (A) Summary of the 532 genes differentially expressed (Student's t-test). (B) Canonical pathways associated with the genes overexpressed in "Immune-High" tumors in the IPC set. Click here for file

Additional file 11 **Figure S2: Correlation of basal breast cancer subgroups (IPC series) and leukocyte cell-type gene expression signatures (GSEA algorithm)**. (A) Results of GSEA with the three tested. NES, normalized enrichment score; FDR, false discovery rate. (B) Enrichment plots for the three significant signatures: T-cell, CD8+ T-cell, and B-cell (from left to right). Click here for file
