# Supplementary material for: Kinome expression profiling and prognosis of basal breast cancers
Source: Mol Cancer. 2011 Jul 21;10:86. doi: 10.1186/1476-4598-10-86 (PMC3156788; doi:10.1186/1476-4598-10-86)
Supplement: Additional file 2 — Supplementary materials. [file 1476-4598-10-86-S2.DOC]

Supplementary Material

Kinome expression profiling and prognosis of basal breast cancers

Renaud Sabatier, Pascal Finetti, Emilie Mamessier,

Stéphane Raynaud, Nathalie Cervera, Eric Lambaudie, Jocelyne Jacquemier,

Patrice Viens, Daniel Birnbaum and François Bertucci

**Gene expression data analysis**

Several types of DNA microarrays were used to generate expression data through the analyzed sets. Before analysis, we mapped hybridization probes across the two technological oligonucleotide-based platforms used across the different sets, Agilent and Affymetrix. Affymetrix gene chips annotations were updated using NetAffx Annotation files ([www.affymetrix.com](http://www.affymetrix.com/); release from 01/12/2008). Agilent gene chips annotations were retrieved and updated using both SOURCE (<http://smd.stanford.edu/cgi-bin/source/sourceSearch>) and EntrezGene (Homo sapiens gene information db, release from 09/12/2008, <ftp://ftp.ncbi.nlm.nih.gov/gene/>). All probes were thus mapped based on their EntrezGene ID. When multiple probes were mapped to the same EntrezGene_ID, the one with the highest variance in a particular dataset was selected to represent the gene retained.

Data sets were then processed as previously described [1]. Briefly, for the Agilent sets, we applied quantile normalization to available processed data. Regarding the Affymetrix sets, we used Robust Multichip Average (RMA) with the non-parametric quantile algorithm as normalization parameter [2]. Quantile normalization or RMA was done in R using Bioconductor and associated packages.

Analysis of each processed data set was then done separately to guarantee the largest number of genes common with the intrinsic gene set and the tested prognostic signatures. The five molecular subtypes were determined using the single sample predictor (SSP) classifier based on a list of 306 intrinsic genes [3]. Briefly, we first identified the genes common between the intrinsic gene set and each data set. We then used Distance Weighted Discrimination (DWD) to normalize data in order to be comparable to the 315 samples of the Hu’s combined test sample set [4]. Next, we defined the expression centroid of each subtype for the common probe sets in this combined test sample set [3]. Finally, we measured the correlation of each sample with each centroid. The sample was attributed the subtype corresponding to the most correlated centroid. To make comparable all data sets, gene expression levels of each set were standardized using the luminal A population as reference. Further analyses only included tumors classified as basal samples.

Other analyses were centered on 771 kinase and kinase-interacting genes, based on an update of the initial kinome description of Manning [5,6]. We matched the 771-gene list with genes available on our Affymetrix U133 Plus 2.0 microarrays. When kinase genes were represented by several probe sets, probe sets with an extension « _at », next « s_at » and followed by all other extensions were preferentially kept. When several probe sets with the best extension were available, the one with the highest median value was retained. Finally, 661 genes were retained (Additional file 3, Table S2).

Analyses were both unsupervised and supervised. Supervised t-test analysis searched for genes upregulated in basal samples compared to at least one of the four other molecular subtypes, with 5% significance and a false discovery rate (FDR) lower than 5%.

DNA microarray data analysis suffers from the dissymmetry of variables with a number of samples inferior to the number of genes being tested. One solution to circumvent this problem is to group genes with correlated and interdependent expression (gene subsets) in a single “metagene”. Metagene expression value is the mean of the normalized expression values of all genes in the respective gene subset. Each metagene is treated as if it were a single gene, thereby reducing data dimension - thus mitigating the multiple testing problem - and smoothing out gene-specific noise through the aggregation within the cluster. This metagene-based approach has been successfully applied by others [7-14]. We defined our metagenes by unsupervised analysis. Unsupervised hierarchical clustering was applied to log_2_-transformed data using the Cluster program using data median-centered on genes, Pearson correlation as similarity metrics and centroid linkage clustering. Results were displayed using TreeView program [15]. We identified robust gene clusters using the quality-threshold (QT) clustering method [15]. The cut-offs for minimal cluster size and minimal Pearson correlation were 15 and 0.6, respectively. A metagene was then computed for each selected cluster, and its prognostic incidence (as continuous value) evaluated using a Cox regression univariate analysis. Metagenes at 5% level significance were selected for further analyses. Metagene prognostic robustness was assessed using resampling method with 100,000 iterations. Random clusters with the same gene number than in the cluster of interest were built, corresponding metagenes were calculated, and their prognostic value was assessed using Cox regression analyses. Once a metagene associated with DFS was defined, its expression value was used to divide the training set into two subgroups then tested for association with DFS. The cut-off was defined as the best threshold dividing the population into two subgroups with the greater DFS difference, ”Metagene-Low” subgroup (expression values inferior to the threshold) and ”Metagene-High” subgroup (expression values above). This cut-off was applied to basal tumors of each validation series, to define the “Metagene-High” and “Metagene-Low” subgroups, which were pooled before prognostic analysis.

For comparison with our classifier, we tested the predictive value of two recently reported classifiers associated with survival in basal BCs: the medullary BC (MBC) classifier [1] which we had defined from a list of genes differentially expressed between basal MBC and non-MBC, and the HER2-derived prognostic predictor (HDPP) [16] associated with survival in both ERBB2+ and basal tumors. The classifiers were applied to each data set separately. We first identified the genes common between the classifier and each set. We then strictly applied to each sample the same classification method (score or Pearson correlation, and scaling methods) as that reported in the original publications. The original cut-off then defined the membership to the predicted good-prognosis group or the predicted poor-prognosis subgroup.

The prognostic metagene and the list of differentially genes were interrogated using Ingenuity Pathway Analysis (IPA) software (Redwood City, CA, USA) to assess significant representation of biological pathways and functions [17]. We also determined if T-cell, CD8+ T-cell, and B-cell signatures [18] were overrepresented in one prognostic subgroup using the gene set enrichment analysis (GSEA) algorithm and 1000 permutations [19].

**Supplementary Material References**

1. Sabatier R, Finetti P, Cervera N, Lambaudie E, Esterni B, Mamessier E, Tallet A, Chabannon C, Extra JM, Jacquemier J, Viens P, Birnbaum D, Bertucci F: **A gene expression signature identifies two prognostic subgroups of basal breast cancer**. *Breast Cancer Res Treat* 2011, **126**: 407-420.
2. Irizarry RA, Hobbs B, Collin F, Beazer-Barclay YD, Antonellis KJ, Scherf U, Speed TP: **Exploration, normalization, and summaries of high density oligonucleotide array probe level data**. *Biostatistics* 2003, **4**:249-264.
3. Hu Z, Fan C, Oh DS, Marron JS, He X, Qaqish BF, Livasy C, Carey LA, Reynolds E, Dressler L, Nobel A, Parker J, Ewend MG, Sawyer LR, Wu J, Liu Y, Nanda R, Tretiakova M, Ruiz Orrico A, Dreher D, Palazzo JP, Perreard L, Nelson E, Mone M, Hansen H, Mullins M, Quackenbush JF, Ellis MJ, Olopade OI, Bernard PS, Perou CM: **The molecular portraits of breast tumors are conserved across microarray platforms**. *BMC Genomics* 2006, **7**:96.
4. Benito M, Parker J, Du Q, Wu J, Xiang D, Perou CM, Marron JS: **Adjustment of systematic microarray data biases**. *Bioinformatics* 2004, **20**:105-114.
5. Manning G, Whyte DB, Martinez R, Hunter T, Sudarsanam S: **The protein kinase complement of the human genome**. *Science* 2002, **298**:1912-1934.
6. Speers C, Tsimelzon A, Sexton K, Herrick AM, Gutierrez C, Culhane A, Quackenbush J, Hilsenbeck S, Chang J, Brown P: **Identification of novel kinase targets for the treatment of estrogen receptor-negative breast cancer**. *Clin Cancer Res* 2009, **15**:6327-6340.
7. Schmidt M, Böhm D, von Törne C, Steiner E, Puhl A, Pilch H, Lehr HA, Hengstler JG, Kölbl H, Gehrmann M: **The humoral immune system has a key prognostic impact in node-negative breast cancer**. *Cancer Res* 2008, **68**:5405-5413.
8. Wright GW, Simon RM: **A random variance model for detection of differential gene expression in small microarray experiments**. *Bioinformatics* 2003, **19**:2448-2455.
9. Rosenwald A, Wright G, Chan WC, Connors JM, Campo E, Fisher RI, Gascoyne RD, Muller-Hermelink HK, Smeland EB, Giltnane JM, Hurt EM, Zhao H, Averett L, Yang L, Wilson WH, Jaffe ES, Simon R, Klausner RD, Powell J, Duffey PL, Longo DL, Greiner TC, Weisenburger DD, Sanger WG, Dave BJ, Lynch JC, Vose J, Armitage JO, Montserrat E, López-Guillermo A *et al*: **The use of molecular profiling to predict survival after chemotherapy for diffuse large-B-cell lymphoma**. *N Engl J Med* 2002, **346**:1937-1947.
10. Pittman J, Huang E, Dressman H, Horng CF, Cheng SH, Tsou MH, Chen CM, Bild A, Iversen ES, Huang AT, Nevins JR, West M: **Integrated modeling of clinical and gene expression information for personalized prediction of disease outcomes**. *Proc Natl Acad Sci USA* 2004, **101**:8431-8436.
11. Potti A, Dressman HK, Bild A, Riedel RF, Chan G, Sayer R, Cragun J, Cottrill H, Kelley MJ, Petersen R, Harpole D, Marks J, Berchuck A, Ginsburg GS, Febbo P, Lancaster J, Nevins JRl: **Genomic signatures to guide the use of chemotherapeutics**. *Nat Med* 2006, **12**:1294-1300.
12. Rody A, Holtrich U, Pusztai L, Liedtke C, Gaetje R, Ruckhaeberle E, Solbach C, Hanker L, Ahr A, Metzler D, Engels K, Karn T, Kaufmann M: **T-cell metagene predicts a favorable prognosis in estrogen receptor-negative and HER2-positive breast cancers**. *Breast Cancer Res* 2009, **11**:R15.
13. Davicioni E, Anderson JR, Buckley JD, Meyer WH, Triche TJ: **Gene expression profiling for survival prediction in pediatric rhabdomyosarcomas: a report from the children's oncology group**. *J Clin Oncol* 2010, **28**:1240-1246.
14. Lenz G, Wright G, Dave SS, Xiao W, Powell J, Zhao H, Xu W, Tan B, Goldschmidt N, Iqbal J, Vose J, Bast M, Fu K, Weisenburger DD, Greiner TC, Armitage JO, Kyle A, May L, Gascoyne RD, Connors JM, Troen G, Holte H, Kvaloy S, Dierickx D, Verhoef G, Delabie J, Smeland EB, Jares P, Martinez A, Lopez-Guillermo A *et al*: **Stromal gene signatures in large-B-cell lymphomas**. *N Engl J Med* 2008, **359**:2313-2323.
15. Eisen MB, Spellman PT, Brown PO, Botstein D: **Cluster analysis and display of genome-wide expression patterns**. *Proc Natl Acad Sci USA* 1998, **95**: 14863–14868.
16. Staaf J, Ringnér M, Vallon-Christersson J, Jönsson G, Bendahl PO, Holm K, Arason A, Gunnarsson H, Hegardt C, Agnarsson BA, Luts L, Grabau D, Fernö M, Malmström PO, Johannsson OT, Loman N, Barkardottir RB, Borg A: **Identification of subtypes in human epidermal growth factor receptor 2--positive breast cancer reveals a gene signature prognostic of outcome**. *J Clin Oncol* 2010, **28**:1813-1820.
17. Ingenuity Pathways Analysis v8.0-2602 (IPA) ([http://www.ingenuity.com](http://www.ingenuity.com/))
18. Palmer C, Diehn M, Alizadeh AA, Brown PO: **Cell-type specific gene expression profiles of leukocytes in human peripheral blood**. *BMC Genomics* 2006, **7**:115.
19. Subramanian A, Tamayo P, Mootha VK, Mukherjee S, Ebert BL, Gillette MA, Paulovich A, Pomeroy SL, Golub TR, Lander ES, Mesirov JP: **Gene set enrichment analysis: a knowledge-based approach for interpreting genome-wide expression profiles**. *Proc Natl Acad Sci USA* 2005, **102**:15545-15550.
