# Supplementary material for: Kinome expression profiling and prognosis of basal breast cancers
Source: Mol Cancer. 2011 Jul 21;10:86. doi: 10.1186/1476-4598-10-86 (PMC3156788; doi:10.1186/1476-4598-10-86)
Supplement: Additional file 9 — Figure S1: Biological network of genes included in or associated with our 28-gene model. A fine-tuning between inhibitor (phosphatases) and activator (kinases) signals regulates lymphocyte anti-tumor immunity. AK and Pyk2 are two of the major kinases that become tyrosine phosphorylated following lymphocyte stimulation. Both are associated to Lck. Lck (lymphocyte specific kinase) and Fyn are cytoplasmic tyrosine kinases of the Src family expressed in T-cells and natural killer (NK) cells, under the T cell receptor (TCR) or Natural cytotoxicity receptor (NCR). Their activity is critical for T and NK cell receptors-mediated signaling, leading to normal T- and NK-cell development and activation. Increased Fyn transcript and protein content in T cells can be observed with high T cell activity. Square 1. LAT is a linker protein essential for activation of T lymphocytes. Its rapid tyrosine-phosphorylation upon TCR stimulation recruits downstream signaling molecules for membrane targeting and activation. LAT is a substrate for Syk/Zap70 kinase and an immediate substrate for both Lck and Syk kinases. Its phosphorylation is an early event leading to T-cell activation. Both Lck and Syk phosphorylate the ITAM-like motifs on LAT, which is essential for induction of the interaction of LAT with downstream signaling molecules such as Grb2, PLC-γ1 and for activation of MAPK-ERK pathways. ZAP70 is thus at the crossroad of several signaling pathways that control lymphocyte development and function and cell survival in response to a wide variety of activator signals coming from the NCR, TCR or other receptor involved in anti-tumor immunity. Square 2. Cytokines receptors express at the membrane also regulate lymphocyte activation through the JAK-STAT signaling pathway. Square 3. In B, T and NK cells, the inhibition of these kinases is mostly mediated by protein tyrosine phosphatases (PTP), regrouping members of the SHP family (SHP-1, SHP-2) or LYP family. These proteins inhibit effector [file 1476-4598-10-86-S9.PPT]

## Slide 1
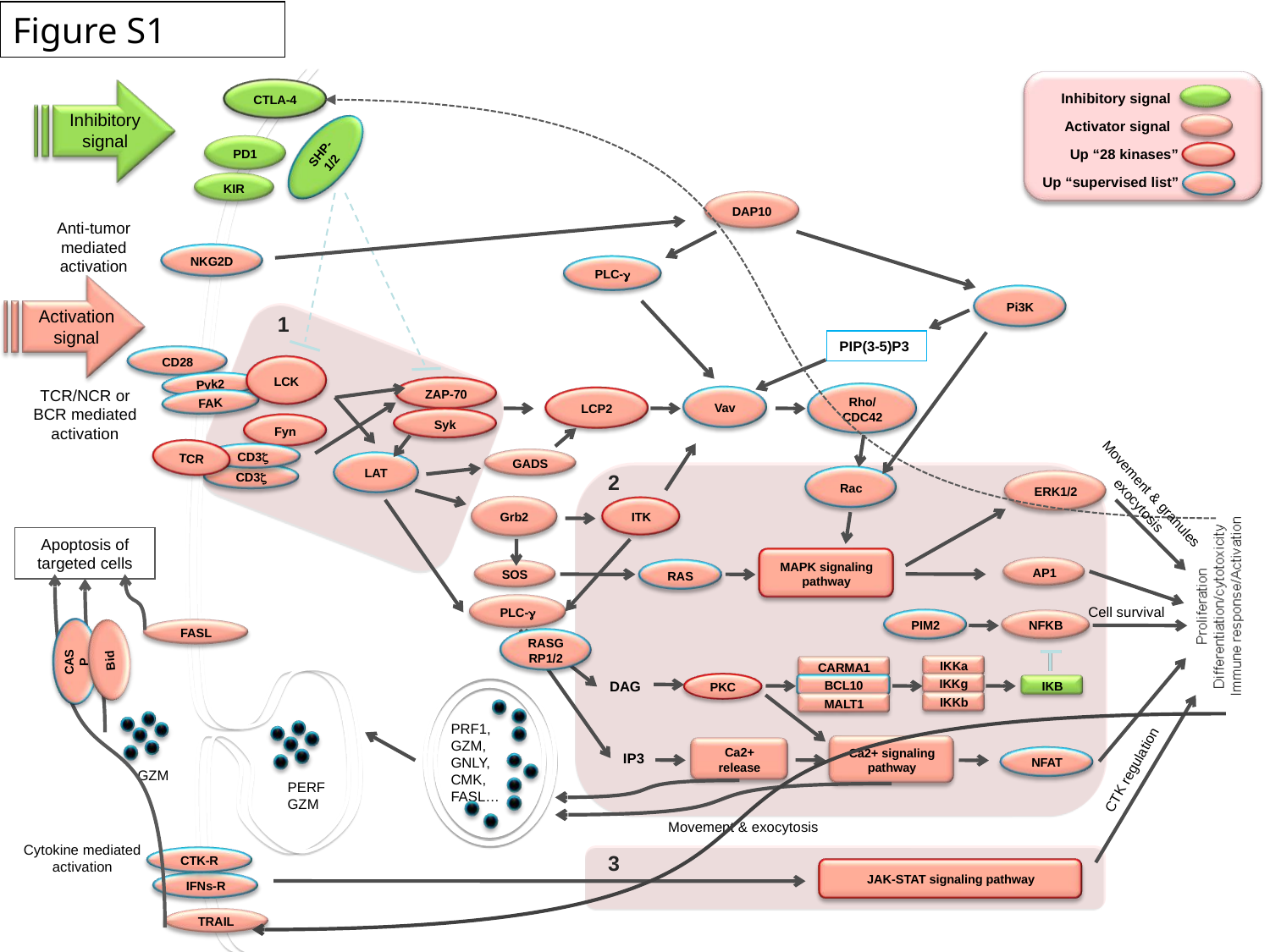

Figure S1
CTLA-4
Inhibitory signal
Inhibitory signal
Activator signal
SHP-1/2
PD1
Up “28 kinases”
Up “supervised list”
KIR
DAP10
Anti-tumor mediated activation
NKG2D
PLC-γ
Pi3K
Activation signal
1
PIP(3-5)P3
CD28
LCK
Pyk2
ZAP-70
TCR/NCR or BCR mediated activation
Rho/ CDC42
Vav
LCP2
FAK
Syk
Fyn
TCR
CD3
GADS
LAT
CD3
Rac
2
ERK1/2
Movement & granules exocytosis
Grb2
ITK
Apoptosis of targeted cells
MAPK signaling pathway
AP1
SOS
RAS
PLC-γ
Cell survival
PIM2
NFKB
CASP
FASL
Bid
RASGRP1/2
IKKa
CARMA1
IKKg
PKC
BCL10
IKB
DAG
IKKb
MALT1
PRF1, GZM,
GNLY,
CMK,
FASL…
Ca2+ signaling pathway
Ca2+ release
CTK regulation
NFAT
IP3
GZM
PERF
GZM
Movement & exocytosis
Cytokine mediated activation
CTK-R
3
JAK-STAT signaling pathway
IFNs-R
TRAIL
