# Supplementary figures and images for: Kinome expression profiling and prognosis of basal breast cancers
Source: Mol Cancer. 2011 Jul 21;10:86. doi: 10.1186/1476-4598-10-86 (PMC3156788; doi:10.1186/1476-4598-10-86)

## Slide 1
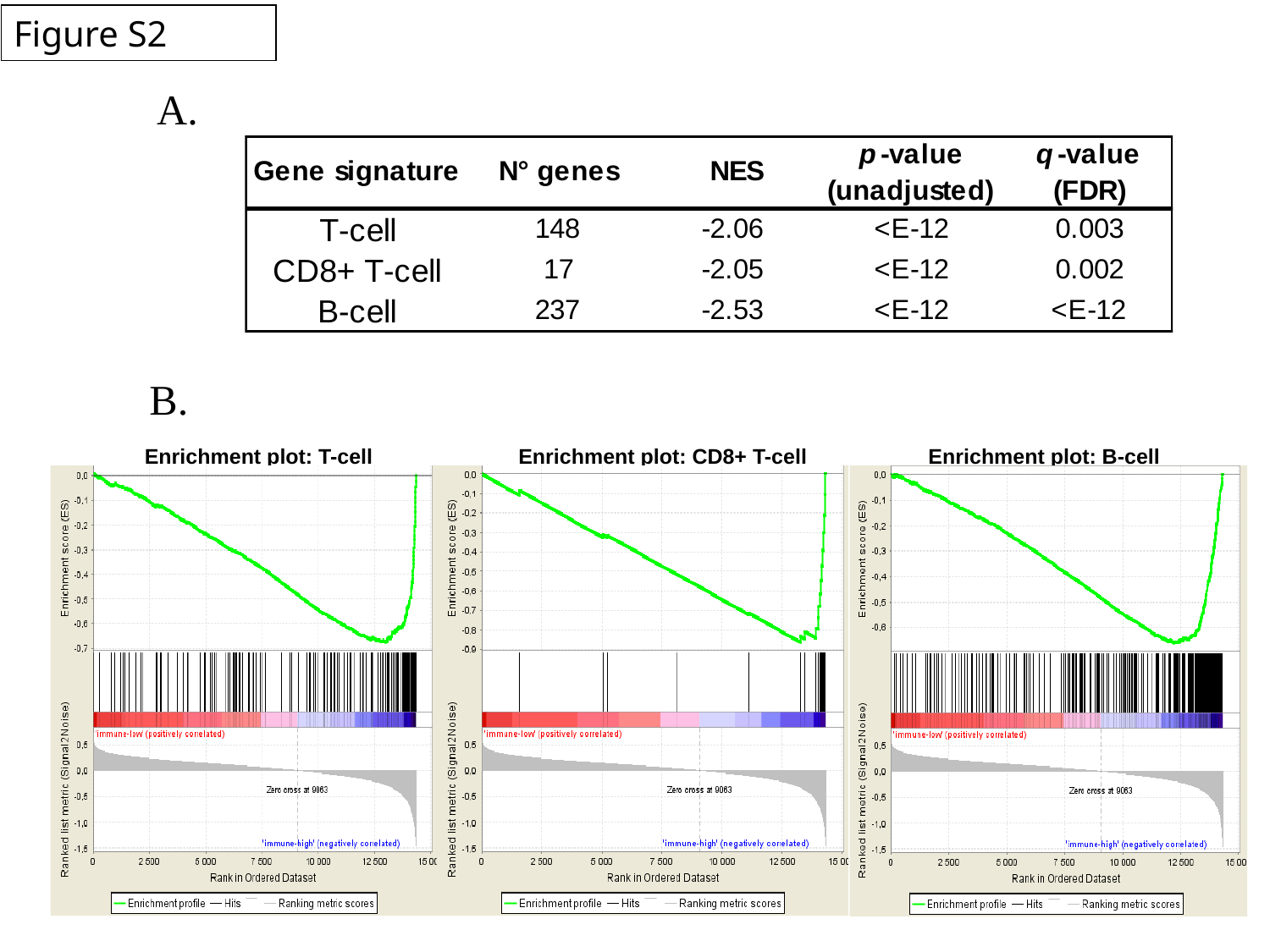

Figure S2
A.
B.
Enrichment plot: T-cell
Enrichment plot: CD8+ T-cell
Enrichment plot: B-cell

Supplement: Additional file 11 — Figure S2: Correlation of basal breast cancer subgroups (IPC series) and leukocyte cell-type gene expression signatures (GSEA algorithm). (A) Results of GSEA with the three tested. NES, normalized enrichment score; FDR, false discovery rate. (B) Enrichment plots for the three significant signatures: T-cell, CD8+ T-cell, and B-cell (from left to right). [file 1476-4598-10-86-S11.PPT]
